# Outbreaks of Acute Gastrointestinal Illness Associated with a Splash Pad in a Wildlife Park — Kansas, June 2021

**DOI:** 10.15585/mmwr.mm7131a1

**Published:** 2022-08-05

**Authors:** Samaria K. Aluko, Syed S. Ishrati, David C. Walker, Mia C. Mattioli, Amy M. Kahler, Kayla L. Vanden Esschert, Kaylee Hervey, Justin Rokisky, Mary E. Wikswo, Joseph P. Laco, Sonalli Kurlekar, Adrienne Byrne, Noelle-Angelique Molinari, Michelle E. Gleason, Christine Steward, Michele C. Hlavsa, Daniel Neises

**Affiliations:** ^1^Division of Foodborne, Waterborne, and Environmental Diseases, National Center for Emerging and Zoonotic Infectious Diseases, CDC; ^2^Oak Ridge Institute for Science and Education, Oak Ridge, Tennessee; ^3^Kansas Department of Health and Environment; ^4^Sedgwick County Health Department, Wichita, Kansas; ^5^Division of Environmental Health Science and Practice, National Center for Environmental Health, CDC; ^6^Division of Viral Diseases, National Center for Immunization and Respiratory Diseases, CDC.

In June 2021, Kansas state and county public health officials identified and investigated three cases of shigellosis (a bacterial diarrheal illness caused by *Shigella* spp*.*) associated with visiting a wildlife park. The park has animal exhibits and a splash pad. Two affected persons visited animal exhibits, and all three entered the splash pad. Nonhuman primates are the only known animal reservoir of *Shigella*. The splash pad, which sprays water on users and is designed so that water does not collect in the user area, was closed on June 19. The state and county public health codes do not include regulations for splash pads. Thus, these venues are not typically inspected, and environmental health expertise is limited. A case-control study identified two distinct outbreaks associated with the park (a shigellosis outbreak involving 21 cases and a subsequent norovirus infection outbreak involving six cases). *Shigella* and norovirus can be transmitted by contaminated water; in both outbreaks, illness was associated with getting splash pad water in the mouth (multiply imputed adjusted odds ratio [aOR_MI_] = 6.4, p = 0.036; and 28.6, p = 0.006, respectively). Maintaining adequate water disinfection and environmental health expertise and targeting prevention efforts to caregivers of splash pad users help prevent splash pad–associated outbreaks. Outbreak incidence might be further reduced when U.S. jurisdicitons voluntarily adopt CDC’s Model Aquatic Health Code (MAHC) recommendations and through the prevention messages: “Don’t get in the water if sick with diarrhea,” “Don’t stand or sit above the jets,” and “Don’t swallow the water.”^†^

On June 18, 2021, the Kansas Department of Health and Environment (KDHE) notified CDC of three cases of shigellosis associated with visiting the wildlife park. Because splash pads are not typically regulated, and thus not inspected, the capacity to identify factors contributing to outbreaks associated with such venues is also limited. KDHE and Sedgwick County Health Department (SCHD) consulted with CDC on the outbreak investigation, which included a case-control study that identified 21 shigellosis cases in respondents who visited the wildlife park on June 11 and six norovirus infection cases in respondents who visited the park 1 week later, on June 18.

## Clinical Laboratory and Epidemiologic Investigation

In Kansas, shigellosis is reportable, and patients are interviewed using standard case investigation forms. The initial investigation identified eight patients with shigellosis who reported entering the splash pad on June 11 and who all experienced signs and symptoms 12–73 hours later. Two of the eight patients had stool cultures positive for *Shigella flexneri* type 1; whole genome sequencing found no base pair differences between the two isolates. The remaining six patients had stool specimens tested using multiplex polymerase chain reaction and were positive for the *Shigella/*enteroinvasive *Escherichia coli* target (*ipaH* gene). Two of these six patients also had specimens that tested positive for the Shiga toxin target (*stx_1_/stx_2_* genes); however, neither stool culture yielded Shiga toxin–producing *E. coli*.

KDHE and the wildlife park, through press releases and Facebook posts, encouraged patrons who visited the park during the period from the seasonal opening day (May 28) through June 19 to voluntarily complete an online outbreak questionnaire during July 12–August 4. The questionnaire requested information on demographic characteristics, activities during wildlife park visits, and signs and symptoms of respondents who experienced gastrointestinal illness (case-patients). Participants who did not experience gastrointestinal illness were considered control respondents.

Summary statistics and bivariate analyses assessed potential exposures. Where item nonresponse was present, missing values were multiply imputed^§^ (*1*,*2*). Unadjusted and adjusted odds ratios and CIs were estimated using Firth penalized maximum likelihood logistic regression (*3*), which can mitigate bias due to rare events. Multiply imputed analyses were compared with complete case analyses. All statistical analyses were conducted in R (version 4.1.3, R Foundation). This activity was reviewed by CDC and was conducted consistent with applicable federal law and CDC policy.^¶^

**FIGURE 1:**
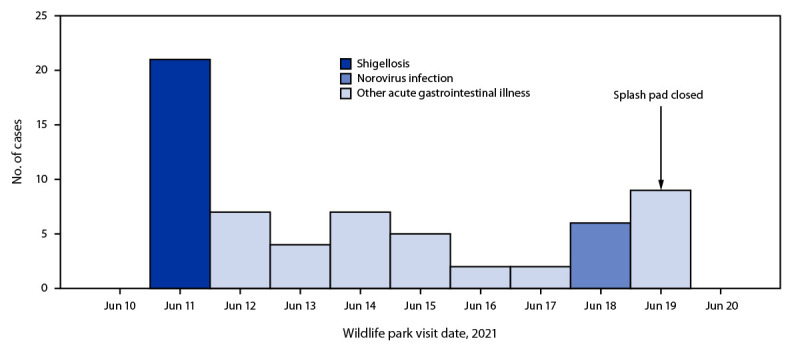
Cases of acute gastrointestinal illness (N = 63)* among study respondents,† by wildlife park visit date — Kansas, June 2021 * A case of shigellosis was defined as diarrhea (three or more loose stools in 24 hours) with onset 12–73 hours after visiting the wildlife park on June 11 (n = 21). A case of norovirus infection was defined as vomiting or diarrhea (three or more loose stools in 24 hours) with onset 12–56 hours after visiting the wildlife park on June 18 (n = 6). A case of other acute gastrointestinal illness was defined as diarrhea (three or more loose stools in 24 hours) with onset 12–73 hours after visiting the wildlife park on June 12, 13, 14, 15, 16, 17, or 19 (n = 36). For these visit dates, no case-patients had clinical laboratory evidence supporting detection of the same pathogen. ^†^ One patient, who entered the splash pad on June 11 and had at least supportive laboratory evidence of *Shigella* detection, did not participate in the case-control study. Two patients, who entered the splash pad on June 18 and had laboratory-confirmed norovirus infection, did not participate in the case-control study.

**FIGURE 2:**
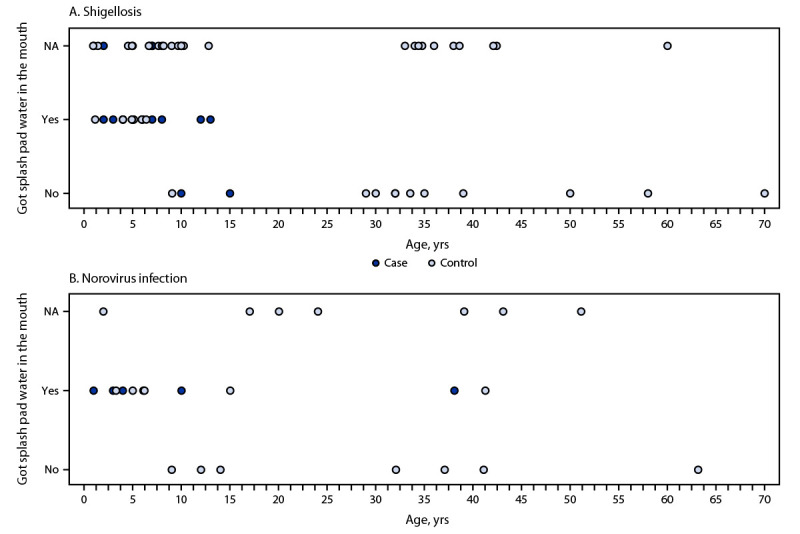
Age distribution of shigellosis (A) and norovirus infection (B) case-patients and control study respondents, by wildlife park visit date* and whether got splash pad water in the mouth— Kansas, June 2021 **Abbreviation: **NA = not applicable. * Shigellosis outbreak associated with park visit on June 11 and norovirus infection outbreak associated with park visit on June 18.

Analysis of data from 404 respondents who only visited the park once during May 28–June 19 identified two distinct outbreaks (Figure 1). Among 72 respondents** who visited on June 11, 21 (29%) experienced illness meeting the shigellosis case definition (three or more loose stools in 24 hours with onset 12–73 hours after visiting the wildlife park on June 11). The median ages of shigellosis case-patients and controls^††^ were 5 years (range = 1–15 years) and 11 years (range = 1–70 years), respectively (p<0.01).^§§^ Both case-patients and controls were predominantly female (62% and 67%, respectively). Playing in the splash pad water was not associated with illness; however, getting splash pad water in the mouth was associated with illness (multivariate complete case adjusted odds ratio [aOR_C_] = 10.2, p = 0.007; aOR_MI_ = 6.4, p = 0.036) (Table) (Figure 2). Touching or feeding lemurs (the only nonhuman primates available for patron interaction) was not associated with illness. Three case-patients were hospitalized for an average of 3 days; no deaths were reported.

**TABLE:** Potential exposures among case-patients and control study respondents who visited a wildlife park — Kansas, June 2021

Potential exposures*^,†,§^	No./Total no. (%)		Bivariate complete case analysis		Multivariate complete case analysis		Bivariate multiply imputed analysis		Multivariate multiply imputed analysis	
	Case patients	Control participants	aOR_C_^¶^ (95% CI)**	P-value	aOR_C_^¶^ (95% CI)**	P-value	aOR_MI_^¶^ (95% CLIP)**	P-value	aOR_MI_^¶^ (95% CLIP)**	P-value
Jun 11, 2021										
*Shigella* ^††,§§^										
Played in splash pad water	21/21 (100)	35/43 (81)	10.3 (1.2–1,356.0)	0.032	—^¶¶^	—^¶¶^	10.3 (1.2–1,356.0)	<0.001	2.6 (0.1–406.2)	0.502
Got splash pad water in the mouth	13/15 (87)	7/19 (37)	9.0 (2.0–56.4)	0.003	10.2 (1.8–109.5)	0.007	10.0 (1.5–83.5)	0.008	6.4 (1.1–65.2)	0.036
Drank water from drinking fountain	3/17 (18)	3/41 (7)	2.7 (0.5–14.0)	0.237	3.0 (0.4–41.9)	0.318	3.3 (0.6–17.1)	0.143	3.1 (0.4–33.4)	0.187
Touched or fed lemurs	4/19 (21)	12/42 (29)	0.7 (0.2–2.3)	0.580	1.2 (0.2–9.0)	0.886	0.8 (0.2–2.5)	0.637	0.9 (0.2–4.6)	0.876
**Jun 18, 2021**										
Norovirus***^,†††^										
Played in splash pad water	6/6 (100)	13/19 (68)	6.3 (0.6–863.0)	0.149	—^¶¶^	—^¶¶^	6.3 (0.6–378.0)	0.149	0.7 (0.003–140.0)	0.866
Got splash pad water in the mouth	6/6 (100)	5/12 (42)	17.7 (1.5–2,501.5)	0.018	24.1 (2.2–4,042.2)	0.007	32.3 (3.0–4,494.9)	0.002	28.6 (2.3–3,584.8)	0.006
Drank water from drinking fountain	0/6 (—)	2/18 (11)	0.5 (0.003–7.5)	0.656	0.1 (0.001–1.8)	0.130	0.42 (0.003–6.0)	0.560	0.1 (0.001–2.0)	0.148

**Abbreviations:** aOR_C_ = adjusted odds ratio, complete case; aOR_MI_ = adjusted odds ratio, multiply imputed; CLIP = combination of likelihood profiles.

* Sex (male or female) was not found to be associated with illness.

^†^ Age was found to be associated with illness but was removed from the model because of its high correlation with getting splash pad water in the mouth.

^§^ Among those who visited on June 11 or June 18, self-reporting indicated 21% (15 of 73) wore the same shoes in the animal area and splash pad; 7% (five of 69) used indoor showers before entering the splash pad, and 59% (24 of 41) always used hand sanitizer or washed hands with soap after touching or feeding animals. None of these behaviors were associated with illness in initial analyses and were removed from consideration before multivariate modeling.

^¶^ aORs calculated using Firth penalized likelihood logistic regression, pooled estimates for multiply imputed analysis.

** CIs calculated using penalized profile likelihood, intervals by CLIP for multiply imputed analysis.

^††^ Case-patients (21) were respondents who experienced diarrhea (three or more loose stools in 24 hours) with onset 12–73 hours after visiting the wildlife park on June 11.

^§§^ Control participants (43) were respondents who did not experience gastrointestinal signs or symptoms (i.e., diarrhea, bloody diarrhea, abdominal pain, nausea, or vomiting) after visiting the wildlife park on June 11.

^¶¶^ Among persons completing responses, all case-patients played in splash pad water; because there was no variation, an aOR for this variable cannot be estimated in the complete case analysis.

*** Case-patients (six) were respondents who experienced vomiting or diarrhea (three or more loose stools in 24 hours) with onset 12–56 hours after visiting the wildlife park on June 18.

^†††^ Control participants (19) were respondents who did not experience gastrointestinal symptoms after visiting the wildlife park on June 18.

Among 27 respondents^¶¶^ who visited the wildlife park on June 18, six (22%) experienced illness meeting the norovirus infection case definition (vomiting or three or more loose stools in 24 hours with onset 12–56 hours after visiting the wildlife park on June 18). Responses for three case-patients indicated a health care provider diagnosed norovirus infection. The median ages of norovirus infection case-patients and controls*** were 5 years (range = 1–38 years) and 20 years (range = 3–63 years), respectively (p = 0.04). Case-patients and controls were predominantly female (83% and 68%, respectively). All six case-patients played in the splash pad water and got the splash pad water in their mouths (Figure 2). Getting splash pad water in the mouth was associated with illness (aOR_C_ = 24.1, p = 0.007; aOR_MI_ = 28.6, p = 0.006). One case-patient was hospitalized for 1 day; no deaths were reported.

## Environmental Microbiology Laboratory and Environmental Health Investigation

The wildlife park’s unregulated splash pad included jets, tipping buckets, and slides. SCHD worked with the splash pad operator and CDC to identify potential factors contributing to the outbreaks. Water stood in the collection tank (into which water drains after spraying users and before it is filtered, disinfected, and resprayed) overnight instead of being continuously recirculated, filtered, and chlorinated. The splash pad did not have an automated controller to measure and help maintain the free chlorine concentration needed to prevent pathogen transmission. In addition, no staff member had documentation of having completed standardized operator training. Splash pad water samples were collected on June 19 from the four sand filters, seven pumps, and collection tank. CDC tested the samples for *Shigella* and fecal indicator bacteria.^†††^ Total coliforms were detected in three of the seven pumps feeding the splash pad features; *E. coli* was detected in one of the coliform-positive pumps.

## Public Health Response

After the splash pad was closed, CDC drafted operation and management guidance for reopening, based on the 2018 MAHC (*4*). SCHD finalized the recommendations and shared them with the splash pad operator. On July 24, the operator reopened the splash pad to the public after voluntarily addressing gaps and implementing best practices, including continuously recirculating, filtering, and disinfecting water (MAHC 5.7.1.1.1 and 5.7.3.1);^§§§^ using an automated controller (MAHC 5.7.3.7.1); and completing operator training (MAHC 6.1.2). After these interventions were implemented, no additional splash pad–associated illnesses were identified.

## Discussion

Pathogens that cause gastrointestinal illness can be transmitted when water contaminated with feces from infected persons is ingested. Splash pads are intended for young children, aged <5 years. Young children are more likely to experience acute gastrointestinal illness including shigellosis and norovirus infection (*5*,*6*), and, because of inadequate toileting and hygiene skills, are more likely to contaminate the water. Swim diapers do not prevent contamination of water (*7*). Children also ingest more recreational water than do adults (*8*). One study of splash pad behaviors documented children wearing diapers, sitting on water jets, and placing their open mouths to the water (*9*). When splash pads jet or spray water, aerosolization depletes the free chlorine concentration, making it more difficult to maintain the minimum concentration necessary to prevent pathogen transmission. These two splash pad outbreaks were caused by pathogens readily inactivated by free chlorine,^¶¶¶^ indicating that the outbreaks might have been prevented by maintaining water disinfection. The environmental health investigation findings elucidated why these outbreaks occurred; no additional cases were identified after the operator implemented reopening guidance, suggesting that pathogen transmission can be prevented.

Splash pads typically do not have standing water in the user area. Thus, they might not meet a jurisdiction’s definition of a treated recreational water venue (e.g., pool) open to the public, which can exempt them from regulation under public health codes. In 1997, the first reported splash pad–associated outbreak also occurred near animals**** (*10*). Although neither the investigation of the 1997 outbreak nor this shigellosis outbreak found evidence of waterborne zoonotic pathogen transmission, they highlight the potential for such transmission. In public animal settings with splash pads, using the splash pad before entering animal areas or changing shoes before entering the splash pad could prevent the introduction and transmission of zoonotic pathogens.

The findings in this report are subject to at least five limitations. First, daily overall wildlife park and splash pad patron counts were not available, and respondent representativeness was not assessed. Second, case-patient and control respondent counts were small, and analyzing data by wildlife park visit date further limited statistical power. However, for visit dates other than June 11 or 18, no case-patients had clinical laboratory evidence supporting detection of the same pathogen. Missing data were multiply imputed to partially address limited statistical power. Third, wording of one response choice lacked clarity^††††^ and might have increased error in exposure observations resulting in increased SE and reduced significance in the association between the splash pad and illness. Fourth, small samples can cause rare events to be overrepresented, leading to bias. Statistical techniques, including Firth penalized logistic regression, were employed to account for this. Finally, the time elapsed from splash pad entry until study response could have led to recall bias.

By identifying factors contributing to outbreaks, environmental health specialists provide data central to developing effective mitigation and prevention strategies for outbreaks associated with treated recreational water venues open to the public. As splash pad use increases, exempting splash pads from regulation under public health codes needs to be reconsidered. CDC’s MAHC recommendations can be voluntarily adopted by U.S. jurisdictions. Environmental health specialists also enforce public health codes through inspections and serve as healthy and safe swimming resources for operators.^§§§§^ To further promote healthy and safe swimming, environmental health specialists can partner with the aquatics sector to engage the public and increase awareness of the importance of healthy swimming steps. Efforts to prevent splash pad–associated outbreaks need to be targeted to caregivers, with prevention messages that include “Don’t get in the water if sick with diarrhea,” “Don’t stand or sit above the jets,” and “Don’t swallow the water.”

**Summary**

**What is already known about this topic?**

Splash pads jet or spray water on users.

**What is added by this report?**

Two outbreaks were associated with a Kansas wildlife park: one caused by *Shigella* and involving 21 cases in study respondents who visited on June 11 and the other caused by norovirus and involving six cases in respondents who visited on June 18. Getting splash pad water in the mouth was associated with illness on both days. Outbreak contributing factors included inadequate disinfection, equipment, and training.

**What are the implications for public health practice?**

Maintaining adequate water disinfection and environmental health expertise and targeting prevention efforts to caregivers of children help prevent splash pad–associated outbreaks.

## Footnotes

* These authors contributed equally to this report.

†
https://www.cdc.gov/healthywater/swimming/swimmers/water-play-areas-interactive-fountains.html

§ Missing values were multiply imputed 60 times using chained equations. CIs were calculated using penalized profile likelihood for complete case analysis and a combination of likelihood profiles for multiply imputed analyses.

¶ 45 C.F.R. part 46.102(l)(2); 21 C.F.R. part 56; 42 U.S.C. Sect. 241(d); 5 U.S.C. Sect. 552a; 44 U.S.C. Sect. 3501 et seq.

** Data were excluded from analysis for respondents when the question asking respondents if they became ill after visiting the wildlife park was unanswered (five); they became ill after visiting the wildlife park but did not know if they experienced gastrointestinal symptoms (two); or the illness was determined to be a secondary case (one). One patient, who entered the splash pad on June 11 and had at least supportive laboratory evidence of *Shigella* detection, did not participate in the case-control study.

†† Controls for shigellosis cases were defined as respondents who did not experience gastrointestinal symptoms (i.e., diarrhea, bloody diarrhea, abdominal pain, nausea, or vomiting) after visiting the wildlife park on June 11.

§§ Case-patient and control age data were compared using the Mann-Whitney U test.

¶¶ Data were excluded from analysis for respondents who became ill after visiting the wildlife park but did not know if they experienced gastrointestinal symptoms (one) or the illness onset date was not reported (one). Two patients, who entered the splash pad on June 18 and had laboratory-confirmed norovirus infection, did not participate in the case-control study.

*** Controls for cases of norovirus infection were defined as respondents who did not experience gastrointestinal symptoms after visiting the wildlife park on June 18.

†††
https://www.standardmethods.org/doi/10.2105/SMWW.2882.194

§§§ For reference purposes, MAHC elements discussed in this report are followed by the specific section number that corresponds to that element.

¶¶¶ At 1 ppm free chlorine, the minimum concentration recommended by CDC and typically required in U.S. jurisdictions for treated recreational water venues open to the public, most pathogens are inactivated within minutes at pH of 7.2–7.8 and temperature of 77°F (25°C). pH will determine the relative amounts of hypochlorous acid (HOCl, a very active chlorine disinfectant) and hypochlorite ion (OCl¯, a less active chlorine disinfectant).

**** In the 1997 Minnesota outbreak, children soaked themselves in the recirculated water by standing on grates above the jets. The jets were 50 yds (46 m) away from ruminants (animal reservoirs of the outbreak etiology, *Cryptosporidium*, a parasite that causes diarrhea).

†††† The question asked if respondents went into the wildlife park splash pad area and provided the following answer choices: 1) Yes, went and played in the splash park, 2) Yes, went to the splash park area but did not play in the water, and 3) No, did not go into the splash park area.

§§§§
https://www.cdc.gov/healthywater/swimming/swimmers/splash-pad-operation-and-management.html
